# Comparison between pipeline embolization device (PED) versus flow redirection endoluminal device (FRED) for intracranial aneurysms: a comprehensive systematic review and meta-analysis

**DOI:** 10.1007/s10143-025-03595-x

**Published:** 2025-06-03

**Authors:** Seyed Farzad Maroufi, Mohammad Sadegh Fallahi, Muhammad Waqas, Othman Bin-Alamer, Manisha Koneru, Joanna M Roy, Jane Khalife, Hamza A. Shaikh, Daniel A. Tonetti

**Affiliations:** 1 Neurosurgical Research Network (NRN), Universal Scientific Education and Research Network (USERN), Tehran, Iran; 2 Department of Neurosurgery, Tehran University of Medical Sciences, Tehran, Iran; 3 Department of Neurosurgery, Jacobs School of Medicine and Biomedical Sciences, University at Buffalo, Buffalo, NY USA; 4 Department of Neurological Surgery, University of Pittsburgh Medical Center, Pittsburgh, PA USA; 5 Cooper Medical School of Rowan University, Camden, NJ USA; 6 Department of Neurological Surgery, Thomas Jefferson University Hospital, Philadelphia, PA USA; 7 Division of the Cooper Neurological Institute, Department of Neurology, Cooper University Health Care, Camden, NJ USA; 8 Division of the Cooper Neurological Institute, Department of Neurosurgery, Cooper University Health Care, Camden, NJ USA

**Keywords:** Pipeline embolization device, PED, Intracranial aneurysms, Flow re-direction endoluminal device, FRED

## Abstract

**Objectives:**

The performance of the Pipeline Embolization Device (PED) and relatively newer double-layered Flow Re-Direction Endoluminal Device (FRED) have been studied for the treatment of intracranial aneurysms, but direct comparisons between PED and FRED are limited. The current systematic review aims at comparing the efficacy and safety of PED and FRED.

**Methods:**

A systematic review of the literature was conducted according to the PRISMA guideline. PubMed, Embase, Scopus, Web of Science, and Cochrane Library were searched, and related records were identified. A meta-analysis of double-arm studies comparing PED and FRED was conducted on angiographic and clinical outcomes, retreatment rates, and complications following treatment.

**Results:**

A total of 15 retrospective double-arm studies, published from 2017 to 2023, were included. Studies were predominantly from the US and Germany. A total of 2231 patients across these studies were analyzed, with 1214 treated using PED and 1017 with FRED. Angiographic outcomes demonstrated no significant difference in occlusion rates between PED and FRED (*P* = 0.35). Retreatment rates trended lower with FRED (*P* = 0.08) but were not significant. Moreover, adjunctive coiling was more frequently utilized with FRED (*P* = 0.04). Complication rates were similar between the two groups. There was no significant difference in mortality between the two devices (*P* = 0.80).

**Conclusion:**

This review provides evidence on the comparable safety and effectiveness of FRED with PED. PED and FRED show comparable angiographic outcomes, with a trend toward lower retreatment rates with FRED. Complication rates and mortality are comparable, with slightly higher historical hemorrhage rate for PED.

## Introduction

The Pipeline Embolization Device (PED) and the Flow Re-Direction Endoluminal Device (FRED) are both flow-diverting stents used in the endovascular treatment of intracranial aneurysms. By rerouting blood flow from the aneurysm sac, these devices promote thrombosis within aneurysm and reconstruction of the parent artery [1]. A number of sizable trials evaluating the safety and efficacy of PEDs have reported high occlusion rates and comparatively low complication rates [2, 3, 4]. The FRED is a newer-generation flow diverter (FD) with a dual-layer stent, consisting of an outer layer designed to divert blood flow and an inner layer to support the construct. Several multicenter experiences have reported acceptable occlusion rates and complication profiles for the FRED device [5, 6, 7]. While both devices aim to achieve similar outcomes, direct comparative studies between PED and FRED are limited. With the increasing number of available FDs, a pooled comparative report is paramount. This systematic review aims to provide a detailed, direct comparison of the performance and safety between these two FDs.

## Materials and methods

The protocol of this review was not previously registered.

### Search strategy

Embase, PubMed, Web of Science, Scopus, and Cochrane Library were searched on February 2024 according to PRISMA guideline, utilizing the following keywords and their equivalents “Pipeline Embolization Device” and “Flow Redirection Endoluminal Device”. A search was also performed on Google Scholar, and the top 200 most relevant results were reviewed. Additionally, a manual search of the bibliographies of the studies included was performed to discover any other pertinent studies.

### Study selection

The inclusion criteria were as follows: (1) various types of clinical studies; (2) double-arm studies comparing the PED and FRED for intracranial aneurysms; (3) providing relevant outcomes such as occlusion, retreatment, complications, and neurological outcomes; and (4) written in English. The criteria for exclusion were: (1) case reports, reviews, letters, and editorials; and (2) studies that did not provide data on outcomes.

### Data extraction

Information was extracted related to different variables, including patient characteristics, procedural specifics, angiographic and clinical outcomes, complications, and retreatment rates. When studies provided matched cohorts, they were categorized into distinct entries for additional analysis based on matching status. The primary outcomes included complete occlusion rates (defined as Raymond-Roy Occlusion Classification [RROC] grade 1, O’Kelly-Marotta [OKM] grade D, or Kamran et al. [8] grade 4), adequate occlusion rates (defined as RROC grade 2, OKM grade C, or Kamran et al. grade 3), and procedure-related complications. Complications analyzed included hemorrhagic events (spontaneous/delayed rupture, intracranial hemorrhage, hemorrhagic stroke, and systemic hemorrhage), thromboembolic events (ischemic stroke, transient ischemic attack, and intraoperative thrombus formation), procedural-related complication (deployment failure or microwire rupture), neurological complications, and in-stent stenosis.

### Risk of bias assessment

The Risk of Bias in Non-Randomized Studies-of Interventions (ROBINS-I) was utilized by two impartial reviewers to assess the bias risk. Any discrepancies were resolved through involvement of a third reviewer.

### Statistical analysis

All analyses were performed using the “meta” package in R (version 4.1.2). Due to the clinical and methodological variations among the studies, random-effect meta-analysis models were used. Median and range data were converted into mean and standard deviation, following Hozo et al. [9]. The functions “metabin” and “metacont” were used to derive effect estimates with the Mantel-Haenszel and DerSimonian-Laird methods [10]. Continuous data was presented using the mean difference, and categorical data was reported using the odds ratio (OR). Leave-one-out analysis was performed to gauge the influence of each study on the final effect. To assess heterogeneity and publication bias, meta-regressions and Egger regression were performed, respectively. A P-value < 0.05 was considered statistically significant.

## Results

### Study selection

**Fig. 1:**
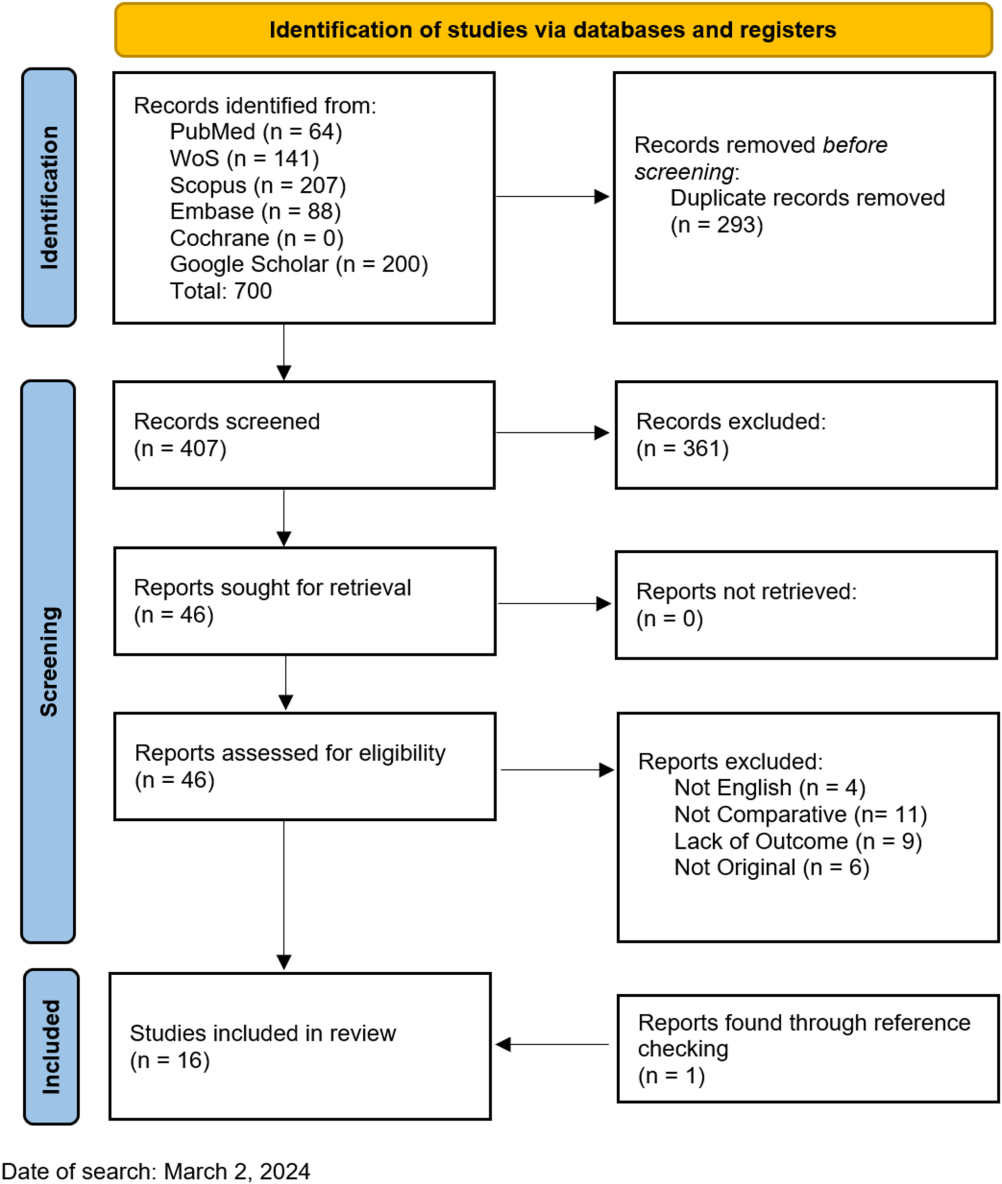
Flow diagram of the screening process

A total of 700 articles were identified on initial search. 407 distinct records were left for abstract screening after duplicate removal, and 46 full-texts were selected for evaluation. Fifteen studies were deemed eligible and were included for analysis. One additional record was identified through reference checking (Fig. 1) [11, 12, 13, 14, 15, 16, 17, 18, 19, 20, 21, 22, 23, 24, 25, 26].

The included studies were retrospective cohorts published from 2017 to 2023 (Table 1). The most common countries of publications were the US and Germany, respectively. Three studies reported data on matched patients in addition to their main sample. Angiographic outcomes were evaluated using various scales across the included studies, with the OKM being the most commonly used scale (6 studies), followed by the RROC (5 studies) and Kamran et al. (2 studies).

**Table 1:** Characteristics of the included studies

Author/Year	Country	Study Duration	FD Type	Imaging Type	Occlusion Criteria	No Patients	Age	Female	Follow-Up	Location	Saccular	Device Type
Bhogal et al., 2018 [10]	Argentina	2009–2018	PED	DSA	RROC	13	56.61	61.54	18.09	ACA (3), PCA (3), PeriC (1), PICA (1), MCA (5)	8	PED
			FRED			5	55.2	100	12	ACA (1), PICA (1), MCA (3)	5	FREDJ
Briganti et al., 2017 [11]	Italy	2008–2015	PED	DSA, MRA, CTA	OKM	31	57	94.12	50	Paraophthalmic ICA (36), PcoA (14), Cavernous ICA (7), ACA (1), MCA (6), BA (2), PICA (1), SCA (2)	67	PED
			FRED			20						FRED
El Naamani et al., 2023 [12]	USA	2018–2021	PED	NR	RROC	115	53.8	85.22	8.8	Anterior Circulation (87)	103	PED
			FRED			35	60.9	85.71	8.4	Anterior Circulation (27)	27	FRED/FRED Jr
Field et al., 2023 [13]	USA	2018–2022	PED	NR	OKM	86	59.3	84.88	20.6	Anterior Circulation (74), Ophthalmic ICA (33.7%), MCA (11,6%), PcoA (25.6%), AcoA (9%)	81	PED/PED Flex/PED Shield
			FRED			33	59.2	66.67	12.4	Anterior Circulation (27), Ophthalmic ICA (18.2%), MCA (11.6%), PcoA (18.2%), AcoA (9%)	31	FRED/FREDX
Griessenauer et al., 2021 [14]	USA, Canada, Austria, China, Germany, Turkey, Iran, Denmark	2012–2019	PED	DSA, CTA	NR	291	57	50.52	12	VA (136), VBJ (19), BA (76), SCA (6), PICA (24), PCA (30)	96	PED Flex
			FRED			84	54	50	24	VA (46), VBJ (2), BA (22), SCA (4), PICA (3), PCA (7)	34	FRED
Griessenauer et al., 2019 [15]	USA, Austria, Turkey, Germany, Denmark,	2013–2017	PED	DSA, CTA	NR	221	60	86.43	8.3	Cavernous ICA (78), Paraophthalmic ICA (352), Other ICA (73)	215	PED/PED Flex
			FRED			282	54.5	78.01	10.8		257	FRED
Gundogmus et al., 2022 [16]	Turkey	2012–2019	PED	DSA	OKM	55	50	85.45	34	Supraclinoid ICA (40), Cavernous ICA (15)	55	PED
			FRED			83	52	86.75	33	Supraclinoid ICA (59), Cavernous ICA (24)	52	FRED
Kunert et al., 2021 [17]	Poland	2009–2016	PED	DSA	OKM	27	NR	NR	NR	Ophthalmic ICA (27)	NR	PED
			FRED			11				Ophthalmic ICA (11)		FRED
Matsukawa et al., 2023 [18]	Japan	2015–2022	PED	DSA, MRA, CTA	RROC	103	64	93.2	12.63	ICA (102), PCA (2)	96	PED
			FRED			93	61	75.27	11.77	ICA (71), PCA (1), MCA (2), VA (14), BA (7)	74	FRED
Michelozzi et al., 2018 [19]	France	2010–2017	PED	DSA	NR	15			18.57	MCA (10), AcoA (5)	NR	PED
			FRED			12			16.91	MCA (8), AcoA (4)		FRED/FREDJ
Patzig et al., 2017 [20]	Germany	2011–2013	PED	DSA	Modified Kamran	17	56.24	47.06	29.67	ICA (12), VA (5)	NR	NR
			FRED			4	49.75	100	20.23	ICA (4)		
Simgen et al., 2023 [21]	Germany	2010–2019	PED	DSA	Kamran	42	51.8	80.95	34.3	ICA (39), VA (3)	40	NR
			FRED			10	51.1	60	31.3	ICA (10)	10	
Soydemir et al., 2023 [22]	Turkey	2013–2019	PED	DSA	OKM	19	57.8	66.67	24.7	MCA	73	PED
			FRED			59				MCA		FRED/FREDJ
Teixeira et al., 2023 [23]	Brazil	2018–2022	PED	DSA	OKM	40	NR	NR	NR	NR	NR	NR
			FRED			196						
Vivanco-Suarez et al., 2022 [24]	USA	2015–2021	PED	DSA, MRA, CTA	RROC	92	62	82.61	14	Anterior Circulation (99), Posterior Circulation (20)	93	PED/PED Flex
			FRED			47	50	85.11	12	Anterior Circulation (57), Posterior Circulation (5)	42	FRED
Pichardo et al., 2020 [25]	Mexico	2016–2019	PED	DSA	RROC	47	61	55.32	12	PCOM (15), Ophthalmic ICA (7), Cavernous ICA (6), Anterior Choroidal (5), Hypophyseal ICA (3), Petrous ICA (4), VA (5), PCA (1), MCA (1)	NR	PED
			FRED			43	61	55.81	12	PcoA (13), Ophthalmic ICA (11), Cavernous (9), Anterior Choroidal (4), Hypophyseal ICA (5), Petrous ICA (2)		FRED

NR: Not Reported; FD: Flow Diverter; PED: Pipeline Embolization Device; FRED: Flow Redirection Endoluminal Device; RROC: Raymond Roy Occlusion Classification; OKM: The O’Kelly-Marotta; DSA: Digital Subtraction Angiography; MRA: Magnetic Resonance Angiography, CTA: Computed Tomography Angiography; ACA: Anterior Cerebral Artery, PCA: Posterior Cerebral Artery, PeriC: Pericallosal Artery, PICA: Posterior Inferior Cerebellar Artery, MCA: Middle Cerebral Artery, PcoA: Posterior Communicating Artery, BA: Basilar Artery, SCA: Superior Cerebellar Artery, AcoA: Anterior Communicating Artery, VA: Vertebral Artery, VBJ: Vertebrobasilar Junction

### Patients characteristics

**Fig. 2:**
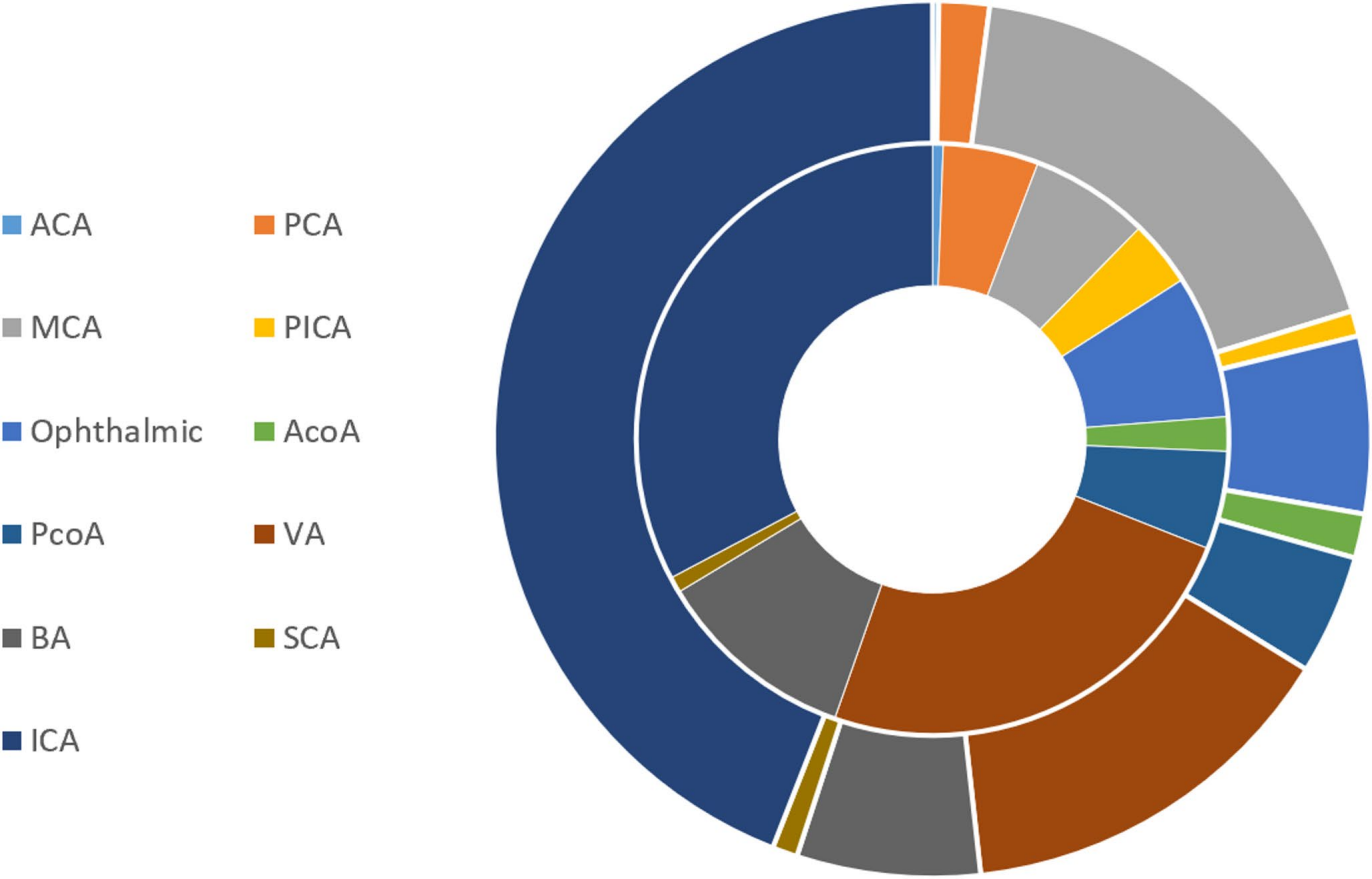
Distribution of various aneurysm location for PED treated (inner circle) and FRED treated (outer circle) patients

A total of 2231 patients were identified across the included studies. 1214 patients (1276 aneurysms) were treated using PED and 1017 patients (1068 aneurysms) were treated with FRED. Patients in the PED group were slightly older than patients in the FRED group (57.42 vs. 55.54 years, *P* = 0.03). The majority of the patients were female in both groups (PED:77.75% vs. FRED:75.59%, *P* = 0.13). Only 2.05% and 0.27% of patients presented with ruptured aneurysms in the PED and FRED groups, respectively (*P* = 0.74). While the majority of aneurysms were saccular, the proportion was slightly higher in the PED group (86.25% vs. 80.52%, *P* = 0.03). More than 90% of the aneurysms were in the anterior circulation (PED:92.02% vs. FRED:96.12%, *P* = 0.96). In studies providing detailed data regarding aneurysm location, there were no significant differences between the two groups in the proportion of posterior cerebral (*P* = 0.33), middle cerebral (*P* = 0.27), ophthalmic (*P* = 0.73), vertebral (*P* = 0.96), and internal carotid artery (*P* = 0.56) locations (Fig. 2). Neck and aneurysm sizes were comparable between the two groups (*P* = 0.66, 0.18, respectively). 14.84% and 9.08% of patients received prior treatment in the PED and FRED groups, respectively (*P* = 0.20). Details of patients’ characteristics are reported in Table 2.

**Table 2:** Meta-analytic details of baseline characteristics, outcomes, and adverse events

Variable	St	PED	FRED	Effect OR/MD	*P*	I^2	H	Eager’s *P*	
Age, years	10	57.42 [55.56; 59.28]	55.54 [54.00; 57.08]	2.36 [0.28; 4.44]	0.03	89.1	3.03	-	
Female, %	11	77.75 [67.64; 85.38]	75.59 [66.21; 83.03]	1.37 [0.91; 2.05]	0.13	50.5	1.42	-	
Follow-Up, months	10	16.62 [14.41; 18.82]	17.44 [12.76; 22.11]	0.66 [-3.37; 4.68]	0.75	98.1	7.19	-	
Rupture Presentation, %	5	2.05 [0.39; 10.08]	0.27 [0.01; 7.99]	1.18 [0.44; 3.18]	0.74	56.7	1.52	-	
Saccular Shape, %	9	86.25 [72.30; 93.78]	80.52 [64.28; 90.48]	1.82 [1.07; 3.11]	0.03	62,7	1.64	-	
Anterior Circulation, %	8	92.02 [57.29; 99.00]	96.12 [66.75; 99.67]	1.02 [0.45; 2.33]	0.96	63.2	1.65	-	
Neck Size, mm	6	4.64 [3.46; 5.82]	4.82 [3.89; 5.75]	-0.14 [-0.75; 0.48]	0.66	88.8	2.99	-	
Aneurysm Diameter, mm (SMD)	12	7.91 [6.92; 8.90]	7.01 [6.21; 7.81]	0.28 [-0.13; 0.68]	0.18	93.0	3.77	-	
Prior Treatment, %	7	14.84 [7.67; 26.76]	9.08 [6.93; 11.81]	1.59 [0.78; 3.23]	0.20	71.0	1.86	-	
Complete Occlusion, %	15	76.02 [64.31; 84.79]	73.95 [58.41; 85.16]	1.21 [0.86; 1.71]	0.27	30.5	1.20	0.79	
Matched Complete Occlusion, %	4	71.78 [61.91; 79.92]	78.36 [65.88; 87.17]	0.79 [0.48; 1.29]	0.35	0.00	1.00	LS	
Adequate Occlusion, %	8	83.46 [77.91; 87.84]	88.46 [78.67; 94.09]	0.73 [0.40; 1.35]	0.32	65.1	1.69	LS	
Matched Adequate Occlusion, %	3	81.61 [73.02; 87.92]	92.71 [75.47; 98.13]	0.49 [0.16; 1.47]	0.20	42.2	1.32	LS	
Residual, %	4	27.84 [3.54; 80.23]	14.06 [7.48; 24.87]	1.00 [0.28; 3.52]	1.00	23.1	1.14	LS	
Adjunctive Coiling, %	7	7.17 [3.58; 13.85]	17.12 [10.20; 27.31]	0.42 [0.18; 0.99]	0.04	71.8	1.88	LS	
mRS < = 2, %	7	93.96 [85.55; 97.61]	97.70 [91.23; 99.43]	0.35 [0.23; 0.54]	< 0.01	0.00	1.00	LS	
Matched mRS < = 2, %	4	91.39 [77.31; 97.07]	99.59 [57.29; 100]	0.31 [0.12; 0.80]	0.01	0.00	1.00	LS	
Complications, %	5	12.52 [7.22; 20.85]	13.25 [5.44; 28.85]	0.94 [0.44; 1.99]	0.86	41.3	1.31	LS	
Hemorrhage^#^, %	11	3.12 [1.73; 5.57]	1.13 [0.61; 2.09]	2.01 [0.94; 4.30]	0.07	0.00	1.00	0.68	
Thromboembolic Complication, %	11	4.74 [2.54; 8.67]	450 [1.74; 11.14]	0.77 [0.49; 1.20]	0.25	0.00	1.00	0.40	
In-Stent Stenosis, %	5	6.52 [0.97; 33.11]	12.11 [2.89; 39.01]	0.60 [0.29; 1.24]	0.17	0.00	1.00	LS	
Neurological Complication, %	6	1.79 [0.94; 3.41]	1.02 [0.35; 2.95]	1.52 [0.52; 4.46]	0.45	0.00	1.00	LS	
Procedural-Related Complication, %	6	6.02 [3.92; 9.15]	4.31 [2.40; 7.62]	1.48 [0.63; 3.48]	0.36	0.00	1.00	LS	
Retreatment^*^, %	6	6.81 [5.20; 8.88]	0.58 [0.04; 8.87]	2.51 [0.90; 7.02]	0.08	25.0	1.16	LS	
Additional Treatment, %	4	5.63 [2.63; 11.65]	4.62 [3.03; 6.97]	1.30 [0.33; 5.16]	0.71	68.5	1.78	LS	
Mortality, %	3	1.48 [0.62; 3.50]	0.79 [0.10; 6.02]	1.21 [0.29; 5.02]	0.80	0.00	1.00	LS	

* Significant on leave-one-out omitting Griessenauer et al., 2021 (*P* = 0.01); # Significant on leave-one-out omitting Gundogmus et al., 2022 (*P* = 0.04); mRS: Modified Rankin Scale; St: Number of studies; OR: Odds Ratio; MD: Mean Difference; SMD: Standardized Mean Diffrernece; LS: Limited number of studies

### Clinical & angiographic outcomes

**Fig. 3:**
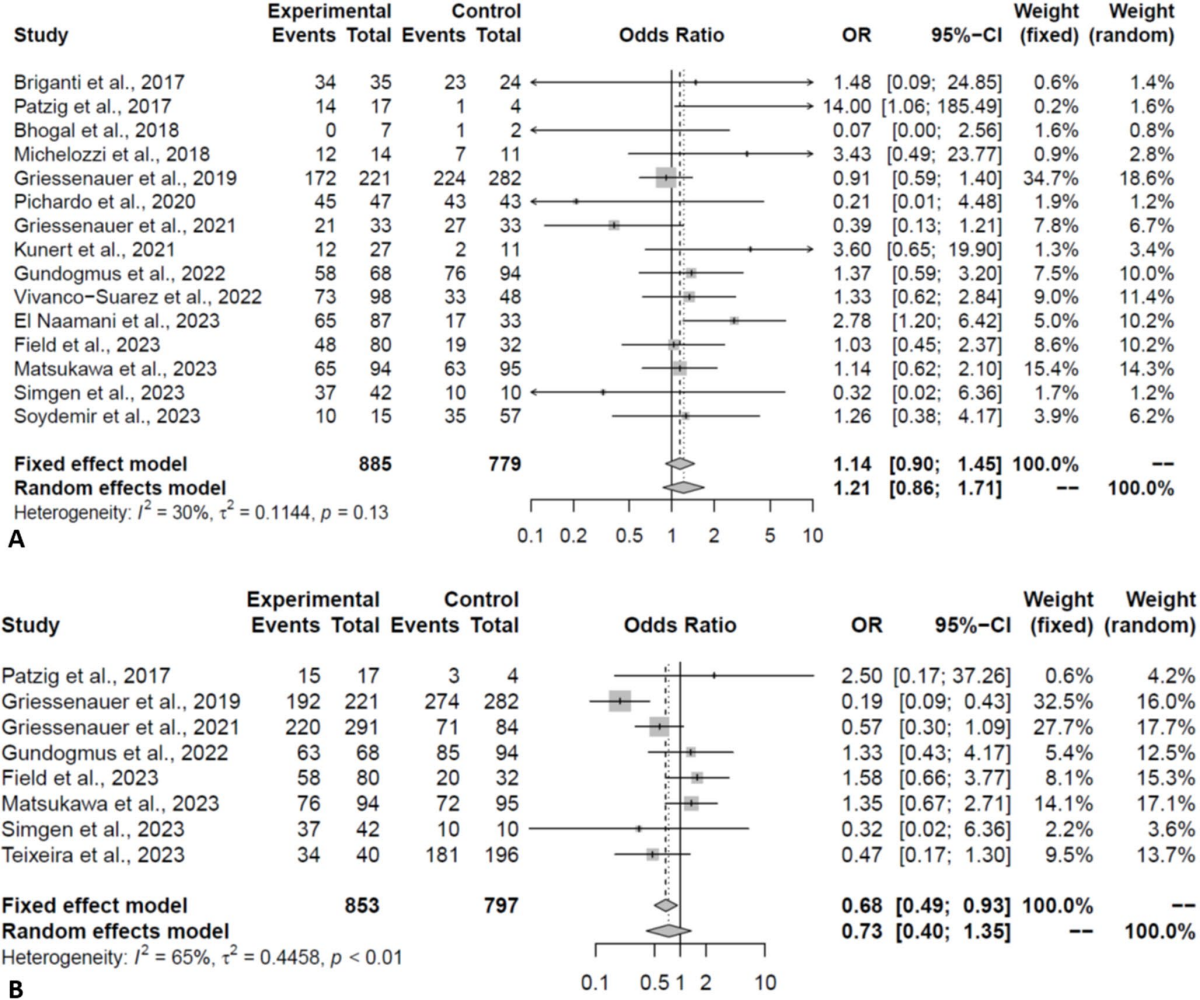
Forest plot depicting the odds of complete (**A**) and adequate (**B**) occlusion in PED vs. FRED groups (Experimental: PED; Control: FRED)

Outcomes are reported in Table 2. The complete occlusion rate was comparable between the two FDs (PED:76.02% vs. FRED:73.95%, OR:1.21, *P* = 0.27, Fig. 3A). The complete occlusion rates were still comparable when only pooling the matched studies (OR:0.79, *P* = 0.35). Similarly, the rate of adequate occlusion (occlusion with small neck/remnant) was comparable in pooling of all (PED:83.46% vs. FRED: 88.46%, OR:0.73, *P* = 0.32, Fig. 3B) and only matched studies (OR:0.49, *P* = 0.20). The residual aneurysm rate (> 50% filling) was comparable between the two groups (OR:1.00, *P* = 1.00). Based on the occlusion scales, the observed effect did not change on subgroup analysis. Moreover, the leave-one-out analysis demonstrated the robustness of the meta-analysis for these outcomes.

While meta-regression demonstrated that female gender, saccular shape, and prior treatment as the main sources of heterogeneity for complete occlusion, only prior treatment was associated with the calculated effect. Regarding adequate occlusion, publication year and follow-up duration were identified as sources of heterogeneity (Table 3). Meta-regression was not performed for residual aneurysm rate due to the limited number of studies.

**Table 3:** Meta-regression details for outcomes with significant heterogeneity on primary analysis

	K	Estimate*10	SE*	*P*-Val	*R*^2
Complete Occlusion					
Year	15	-0.48	8.20	0.95	0.00
Age	13	-2.83	5.92	0.63	0.00
Female %	13	2.52	1.64	0.13	32.96
Follow-Up	14	0.62	2.08	0.77	0.00
Rupture Rate %	13	-2.92	2.58	0.26	3.01
Saccular Shape %	11	1.46	1.02	0.15	34.23
Prior Treatment %	8	3.70	1.75	0.03	100.00
Additional Coiling %	9	1.27	1.51	0.40	0.00
Aneurysm Size	11	-0.18	9.26	0.98	0.00
Adequate Occlusion					
Year	8	8.12	12.59	0.52	5.67
Age	7	4.13	10.25	0.69	0.00
Female %	7	0.50	3.03	0.87	0.00
Follow-Up	7	4.13	4.08	0.31	8.86
Rupture Rate %	7	0.40	4.29	0.93	0.00
Saccular Shape %	6	0.12	1.96	0.95	0.00
Prior Treatment %	4	7.53	8.10	0.35	0.00
Additional Coiling %	5	1.35	4.39	0.76	0.00
Aneurysm Size	4	19.75	27.23	0.47	0.00
Need for Adjunctive Coiling					
Year	7	27.12	18.89	0.15	23.79
Age	6	8.93	12.52	0.48	0.00
Female %	6	10.33	16.21	0.52	0.00
Follow-Up	7	1.40	5.40	0.80	0.00
Rupture Rate %	6	-7.60	10.72	0.48	0.00
Saccular Shape %	6	-4.40	7.26	0.54	0.00
Prior Treatment %	5	-2.69	5.18	0.60	0.00
Aneurysm Size	5	2.86	20.78	0.89	0.00
Retreatment					
Year	6	4.26	24.20	0.86	0.00
Age	6	15.29	30.00	0.61	0.00
Female %	6	4.87	2.58	0.05	100.00
Follow-Up	6	-3.49	7.93	0.66	0.00
Rupture Rate %	6	-7.41	4.15	0.07	100.00
Saccular Shape %	6	2.76	1.43	0.05	100.00
Prior Treatment %	4	-0.11	11.85	0.99	0.00
Additional Coiling %	4	3.25	16.19	0.84	0.00
Aneurysm Size	4	16.62	31.27	0.60	0.00

**Fig. 4:**
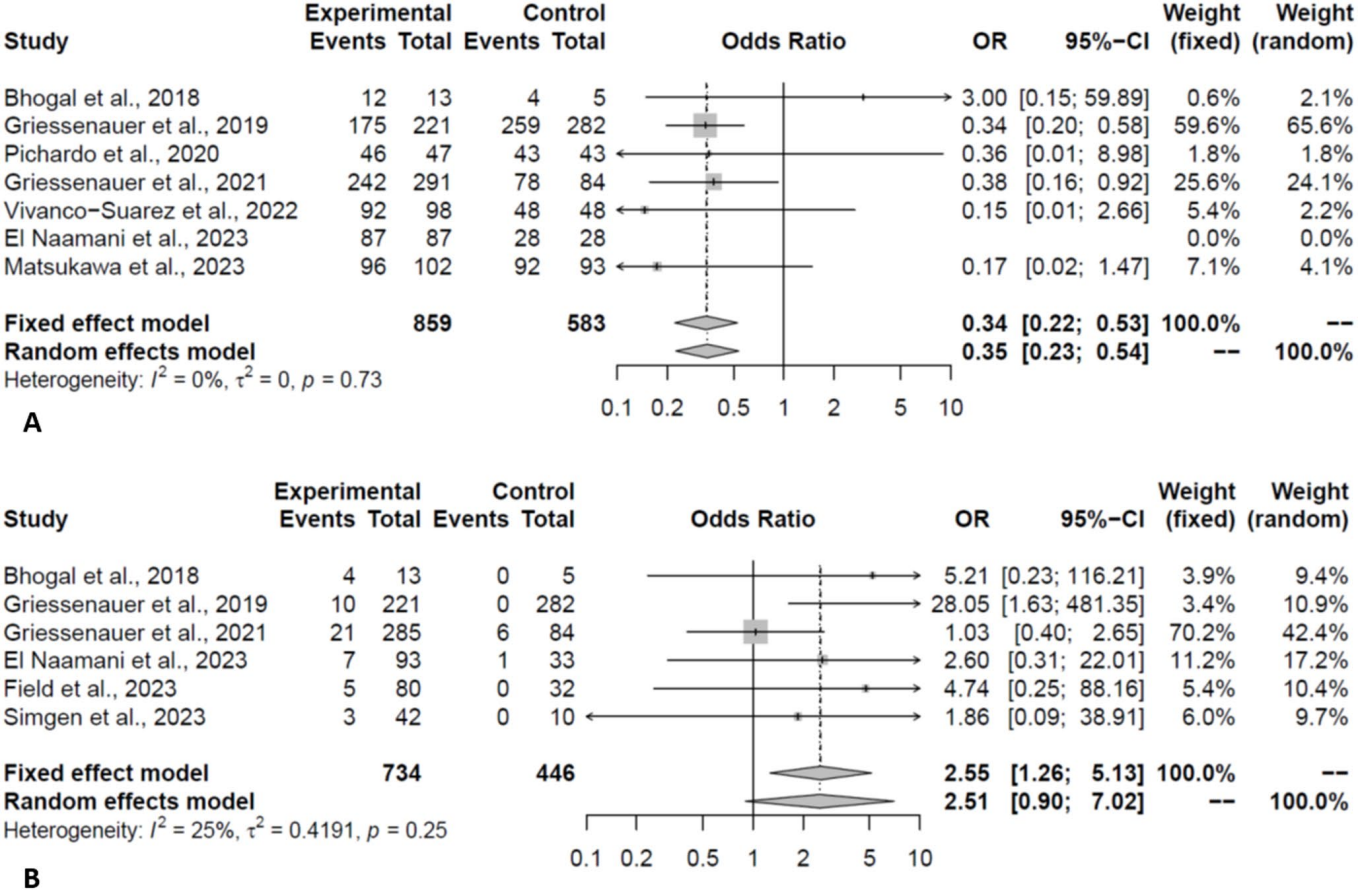
Forest plot depicting the odds of good functional outcome (**A**) and retreatment (**B**) in PED vs. FRED groups (Experimental: PED; Control: FRED)

Good functional outcome (mRS ≤ 2) at the last follow-up (1447patients) was more common in the FRED group compared to the PED group (17.12% vs. 7.17%, OR:0.35, *P* < 0.01, Fig. 4A). Analysis of matched studies revealed a similar effect (OR:0.31, *P* = 0.01). The observed effect did not change on leave-one-out analysis.

Adjunctive coiling was more frequently required in patients treated with FRED (17.12% vs. 7.17%, OR:0.42, *P* = 0.04). Moreover, while there was a trend toward lower retreatment rates in the FRED group, retreatment rates were not significantly different (PED:6.81% vs. FRED:0.58%, OR:2.51, *P* = 0.08, Fig. 4B). However, the difference in retreatment rates was significant on leave-one-out analysis, after omitting Griessenauer et al. (OR:4.71, *P* = 0.01). The main source of heterogeneity was the year of study for adjunctive coiling. Regarding retreatment, the heterogeneity was sourced from the female gender, rupture rate, and saccular shape (Table 3).

### Complications & mortality

**Fig. 5:**
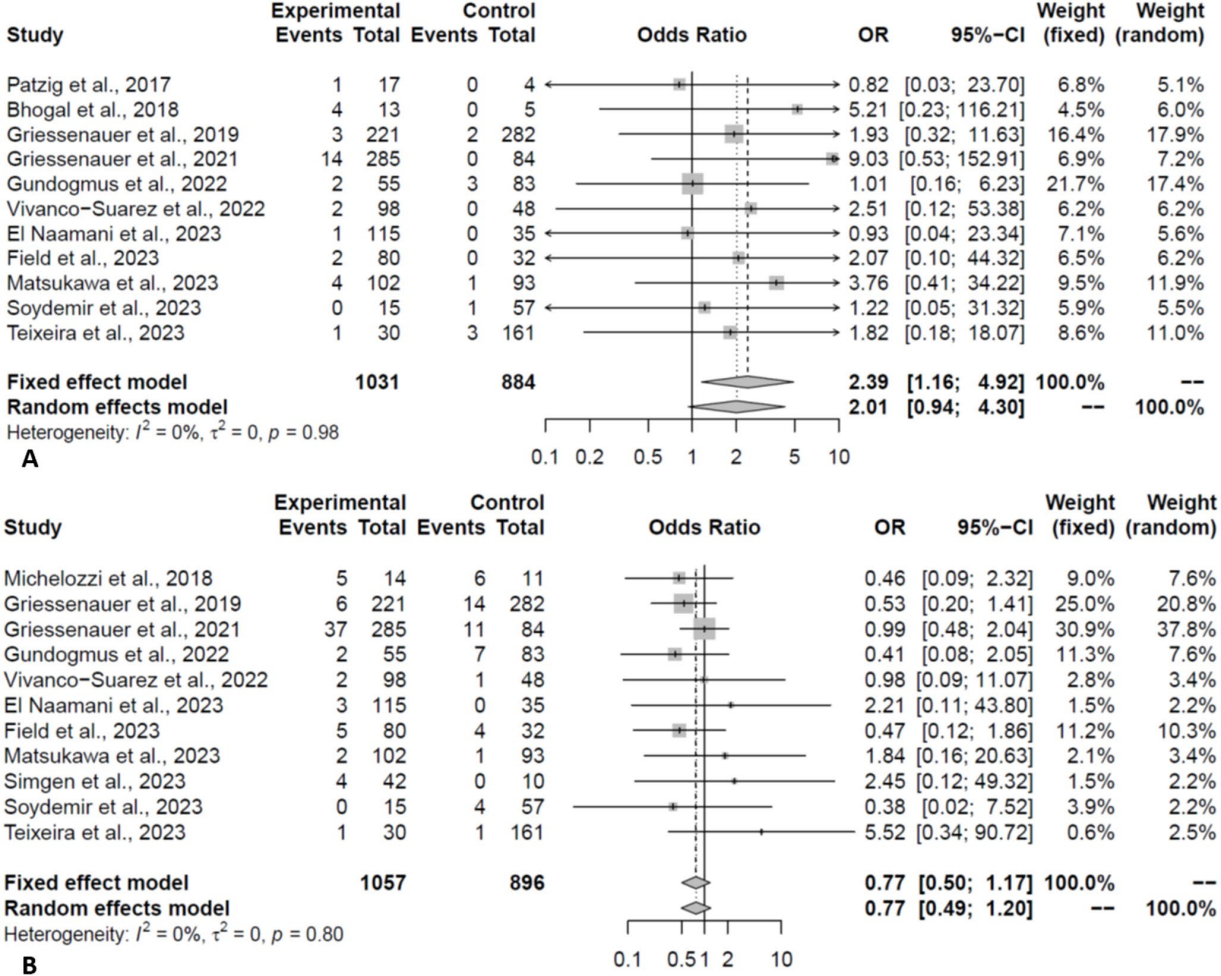
Forest plot depicting the odds of hemorrhage (**A**) and thromboembolic (**B**) events in PED vs. FRED groups (Experimental: PED; Control: FRED)

**Fig. 6:**
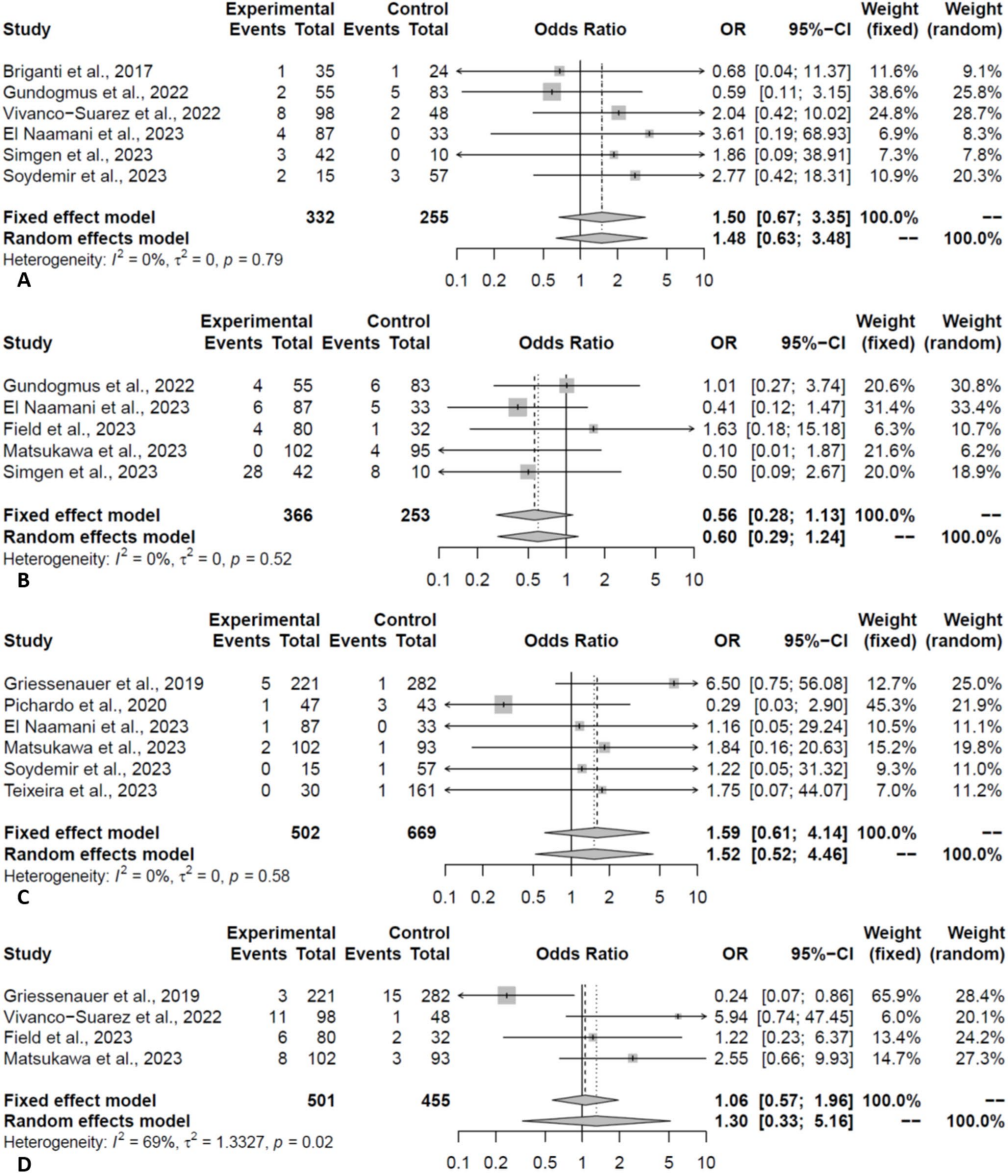
Forest plot depicting the odds of procedural-related complications (**A**), in-stent stenosis (**B**), neurological complications (**C**), and need for additional treatments (**D**) in PED vs. FRED groups (Experimental: PED; Control: FRED)

The two FDs had comparable total complication rates (PED:12.52% vs. FRED:13.25%, OR:0.94, *P* = 0.86). Meta-regression of total complication was not conducted due to the limited number of studies. The hemorrhage rate was 3.12% and 1.13% in the PED and FRED groups, respectively (OR:2.01, *P* = 0.07, Fig. 5A). In the leave-one-out analysis, the hemorrhage rate was significantly higher in the PED group, when omitting Gundogmus et al. (OR:2.35, *P* = 0.04). Rates of thromboembolic complication were comparable between the two groups (PED:4.74% vs. FRED:4.50%, OR:0.77, *P* = 0.25, Fig. 5B). Procedural-related complication rates were comparable between the two groups (PED:6.02% vs. FRED:4.31%, OR:1.48, *P* = 0.36, Fig. 6A). In-stent stenosis was observed in 6.52% and 12.11% of patients in the PED and FRED groups, respectively (*P* = 0.17, Fig. 6B).

Neurological complication rates were comparable between the two groups (PED:1.79% vs. FRED:1.02%, OR:1.52, *P* = 0.45, Fig. 6C). The need for additional treatment (e.g. balloon angioplasty, etc.) was comparable between the two groups (PED:5.63% vs. FRED:4.62%, OR:1.30, *P* = 0.71, Fig. 6D). Lastly, mortality rates were comparable between the two groups (PED:1.48% vs. FRED:0.79%, OR:1.21, *P* = 0.80).

### Risk of bias

**Fig. 7:**
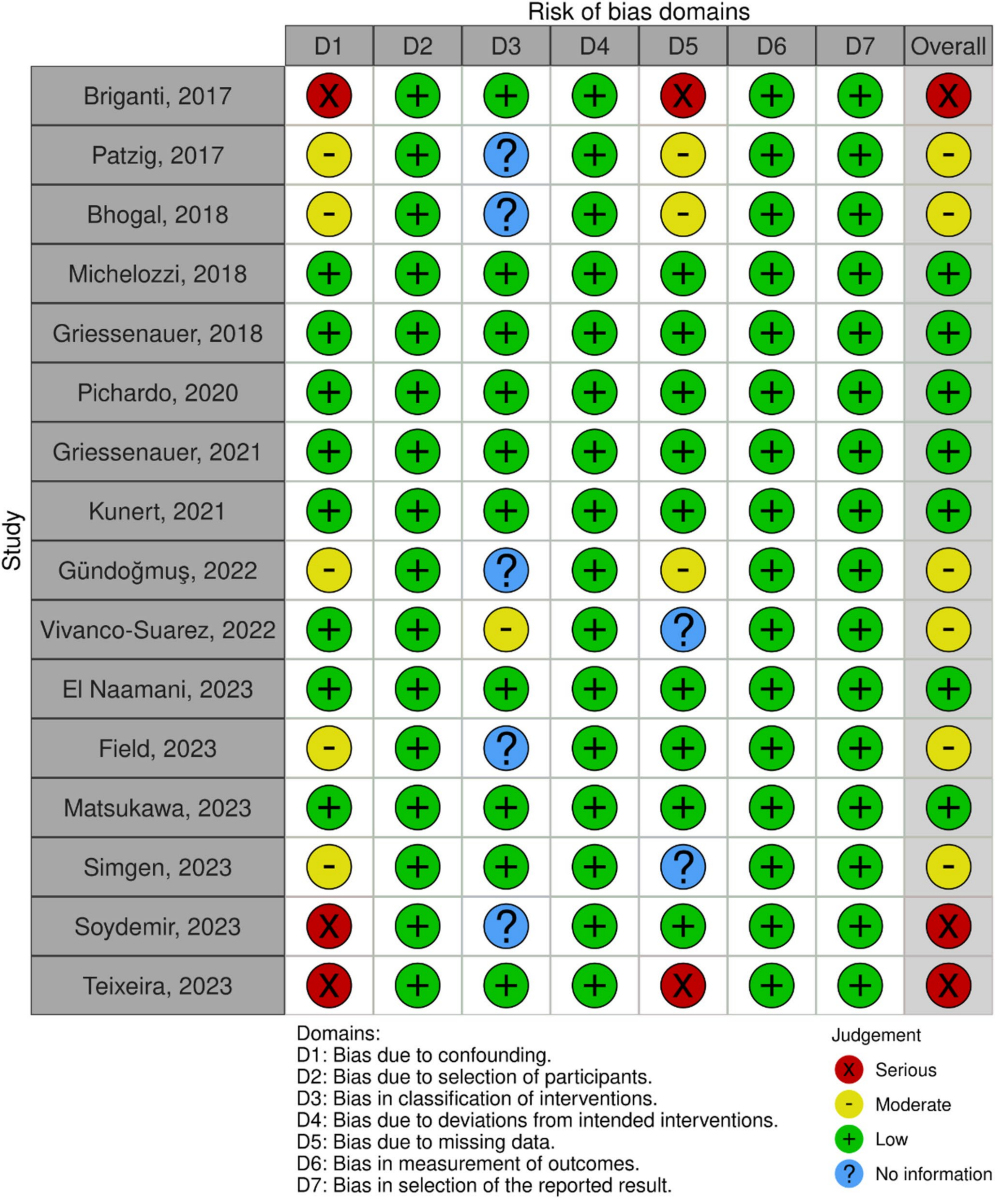
Details of risk of bias according to ROBINS-I

The majority of studies had low to moderate risk of bias (81.25%) with confounding factors being the main source of bias (Fig. 7). None of the analyzed outcomes revealed publication bias on Egger’s regression and visual inspection of funnel plots.

## Discussion

### Overview

FDs, placed in the parent artery, induce stasis within the aneurysm sac by diverting blood flow. Over time, endothelialization around the neck results in complete exclusion of the aneurysm from the vasculature [27]. Two FDA-approved FDs, PED and FRED, offer solutions for complex aneurysms that are often difficult to treat with traditional methods [7]. PED utilizes a cylindrical mesh design, featuring platinum and cobalt-chromium braided strands, available in various single or multi-layered configurations. In contrast, FRED is a braided double-stent device made from nitinol wires, with expansions of the outer stent at both ends.

Although both PED and FRED have shown promising effectiveness and safety, there are controversies regarding the comparison between the two devices. While a recent meta-analysis compared these two approaches, it suffered from incomplete inclusion and severe statistical shortcomings [28]. Accordingly, this is the most comprehensive review comparing PED and FRED and factors associated with various outcomes while considering the baseline characteristics and matching status of the included studies. The results reveal comparable occlusion rates and safety profiles for the two devices. Interestingly, FRED was associated with both an increased need for adjunctive coiling and better functional outcomes following treatment, which potentially suggests the baseline functional status was better in the FRED group. Given the extensive meta-analysis and minimal heterogeneity of calculated effects, these findings provide strong evidence of the non-inferiority of the FRED device compared to the more commonly utilized PED.

### Angiographic and clinical outcome

PED was approved by FDA for treating large/giant, wide-necked aneurysms in the internal carotid artery, specifically from the petrous to the superior hypophyseal segments in 2011. This approval was based on the results of the PUFS trial which demonstrated a high rate (73.6%) of complete occlusion without major narrowing of the parent artery or need for additional embolic material at 180-day follow-up [29]. Further follow-up data from the PUFS trial showed increasing complete occlusion rates, reaching 95.2% at 5 years for this challenging cohort of aneurysms. Importantly, the rates of new serious adverse events were low, at 1%, 3.5%, and 0% at 1, 3, and 5-years, respectively [30, 31]. Consistently, the PREMIER study reported a complete occlusion rate of 76.8% at 1-year and 83.3% at 3-year evaluation [32, 33]. Due to the high efficacy and favorable safety profile of PEDs, further technological advancements have been made to improve ease of use and reduce complications. These advancements include the introduction of PED Flex with reheating technology and PED Flex with Shield technology [34, 35, 36]. Both iterations have shown comparable occlusion rates to the original PED while potentially reducing complication rates [37, 38].

The first large study of the FRED device, involving 15 European neurovascular centers, found that complete occlusion rates increased over time: 20% at 90 days, 82.5% at 180 days, 91.3% at 1 year, and 95.3% for follow-ups exceeding 1 year. Additionally, temporary and permanent complications occurred in 3.2% and 0.8% of procedures, respectively, with an overall mortality rate of 1.5% [39]. Further evidence came from a US pivotal trial focusing on FRED for intracranial aneurysms considered unsuitable for traditional endovascular treatments. This trial showed a technical success rate of 57.6% at 1 year. Additionally, 6.2% of patients experienced death or major stroke within 30 days, or any major stroke or neurological death affecting the same side of the body within 12 months of the procedure [40]. A post-market evaluation of FRED showed similar findings: 74.2% complete occlusion, 11.3% near-complete occlusion, 2.8% retreatment rate, and 2.8% permanent ischemic complications [6]. The present study found consistent angiographic occlusion rates with no significant differences between the PED (complete:76.02%, adequate:83.46%) and FRED (complete:73.95%, adequate:88.46%). Several studies reviewed here report comparable occlusion rates between FRED and PED. However, Matsukawa et al. found a significantly higher rate of complete and adequate occlusions with FRED compared to PED. They suggested this could be due to the space between FRED’s layers, which disrupts blood flow, increases stagnation, traps platelets, and promotes further clot formation [41]. It should be noted that PED received FDA approval approximately eight years before FRED and surgeons are more experienced in using PED [37]. However, as demonstrated in the present analysis, both methods demonstrate similar effectiveness, even after matched analysis, suggesting that the lessons learned from operators experienced with PED deployment may be relevant to the use of the FRED. Theoretical technical advantages to FRED might include a self-expanding structure, easier opening in landing zones (smoother deployment in both the distal and proximal ends of the device), and a smaller size of delivery catheter required for FRED Jr in treating aneurysms in smaller and distal arteries, potentially offering greater versatility [27].

Furthermore, regarding neurological outcomes after treatment, our study found that patients who received FRED had significantly higher rates of favorable outcomes compared to those who received PED in the published comparative studies. This finding remained significant even when we restricted the analysis to matched studies only. Although the significant difference may have been attributed to the lower rates of hemorrhagic complications, the reason for this observed difference is not yet fully understood. It is possible that selection bias and other factors not accounted for in the matching process may have influenced the results, and it is possible that the FRED was used in patients with better baseline functional status, or in easier-to-treat aneurysms [15]. Further comparative studies are needed to elucidate these findings.

### Procedural details

The use of adjunctive coiling alongside FDs for treating intracranial aneurysms is a topic of debate. Some studies suggest that adjunctive coiling can promote progressive intra-aneurysmal thrombosis, leading to improved aneurysm occlusion, and potentially preventing delayed rupture [42]. Additionally, it might be more effective in inducing early complete occlusion and may be more suitable for cases with high rupture risk [43, 44, 45]. However, a meta-analysis revealed comparable occlusion rates between patients receiving FDs with or without adjunctive coiling [46]. Our study revealed significantly higher rates of adjunctive coiling in the FRED group compared to the PED group. This could be due to specialists favoring FRED for patients deemed to have a higher risk of rupture, a profile often requiring adjunctive coiling. Interestingly, the meta-regression analysis showed no significant association between adjunctive coiling rates and either occlusion or complication rates. These findings align with the aforementioned meta-analysis, suggesting that adjunctive coiling might not significantly impact outcomes in either FRED or PED treatment.

### Complications

Our study identified a trend toward increased hemorrhagic complications in patients treated with the PED, which became statistically significant after excluding the study by Gündoğmuş et al. Their study reported higher hemorrhage rates in the FRED group compared to other included publications. This discrepancy may be attributable to the higher proportion of ruptured aneurysms (8% vs. 4%) and significantly greater use of adjunctive coiling (37% vs. 22%) in their FRED cohort relative to the PED group in the same study, both of which suggest a higher risk of re-rupture and have known associations with hemorrhagic complications following flow diversion therapy [47, 48]. The difference in hemorrhagic complication between PED and FRED may not be solely due to the devices themselves. Existing research supports this notion. For instance, Gündoğmuş et al. reported various causes of hemorrhage unrelated to device type, including rupture of a previously treated aneurysm, bleeding caused by medication, spontaneous rupture despite coiling, inadequate coiling technique, and device migration [17]. Furthermore, the dual-layer architecture of FRED may offer advantages in directing blood flow away from the aneurysm while maintaining circulation in nearby perforating vessels. This design has the potential to mitigate ischemic events and hemorrhages. Additionally, the extent of flow diversion could be influenced by the surface area coverage of the aneurysm neck by the device’s struts. With FRED’s inner layer potentially providing greater metal coverage, there could be a more rapid occlusion of the aneurysm, potentially resulting in reduced hemorrhage rates.

Thromboembolic events are among the most serious complications of FDs, occurring in approximately 6% of patients [49, 50]. In our study, the pooled rates of thromboembolic events were 4.7% in the PED group and 4.5% in the FRED group. These rates vary across studies, likely due to differences in methodology and patient populations. Previous single-arm studies reported thromboembolic complication risks ranging from 2 to 7% following PED placement, aligning with our findings [33, 51, 52]. However, a recent study observed a thromboembolic event rate of 10.71% in patients treated with first-generation PED or PED Flex, compared to 3.57% for the PED Shield [53]. Similarly, thromboembolic event rates for FRED vary significantly between studies, [54] with one reporting a 6.9% incidence [5]. The higher thrombogenicity of FRED compared to other FDs has been corroborated by in vitro studies, potentially attributable to its dual-layer design. This structure increases metal surface area and creates localized turbulent blood flow, promoting platelet activation, fibrin deposition, and thrombus formation [55]. Notably, FRED X—a newer surface-coated device—demonstrated a markedly lower ischemic event rate compared to its predecessor (0.6% vs. 5.1%, respectively) [56]. Discrepancies between studies may arise from variations in patient selection criteria, antiplatelet regimens, medication adherence, clopidogrel resistance, procedural learning curves, and follow-up duration. Additional randomized controlled trials are warranted to validate and expand upon these findings.

Previous research suggests a link between the double-layer structure of FRED and a higher incidence of in-stent stenosis compared to PED. This association is hypothesized to be due to potential blood flow disruption and stasis within the dual layers, triggering platelet activation, fibrin buildup, and ultimately, clot formation [13, 55, 57]. However, in-stent stenosis rates were comparable between the two devices in our study. Additionally, we found nearly significantly higher retreatment in PED, which reached statistical significance after excluding the study by Griessenauer et al. [15]. This discrepancy arose because their study reported elevated retreatment rates following the FRED, contrasting with four other studies where no patients in the FRED group required retreatment [14, 16, 22, 58]. The Griessenauer et al. [15]. study included patients with posterior circulation aneurysms treated with PED or FRED as off-label interventions. The higher retreatment rates for FRED in their cohort may reflect the clinical teams’ heightened vigilance in detecting and managing incomplete occlusion or recurrence. This could stem from the inherent challenges of using FDs in posterior circulation aneurysms, compounded by limited institutional experience with FRED at the time of the study. Several factors may influence the difference in retreatment rates including a higher prevalence of incomplete occlusion with PED and a lower bar for retreatment decisions by the physicians managing those cases [16]. Overall, our findings suggest similar safety profiles for the two FDs when considering adverse events. However, future randomized controlled trials could provide more definitive insights.

In our present study, we found that the rates of additional treatment were comparable between the two groups. This observation aligns with the similarity of adverse events, including in-stent restenosis, hemorrhage, and thromboembolic events. However, a study by Matsukawa et al. revealed significantly higher rates of additional treatment in the PED treated patients [41]. This discrepancy may be ascribed to technical differences in performing additional treatment using stents and balloon angioplasty. For example, while previous research has demonstrated the safety and effectiveness of balloon angioplasty for foreshortening and improving apposition of PED, [59] in the case of FRED, passing a balloon through the stent is challenging due to its double-stent structure. The inner mesh of FRED may hinder the navigation of the balloon through the entire length of the stent [60].

### Limitations

This study has several limitations to consider. While both PED and FRED are FDA-approved for treating large, wide-necked intracranial aneurysms, the comparative studies primarily focused on saccular aneurysms. This limits the applicability of our findings to the officially intended uses of PED and FRED. A limited number of studies provided details on the specific scales used to assess aneurysm occlusion. This prevented us from conducting analyses on specific occlusion levels. Two studies had slight overlap in their cohorts but were included to prevent patient loss. There was also significant heterogeneity across the studies in terms of patient selection criteria, device types, and follow-up durations, all of which could potentially influence the outcomes of our analysis. Furthermore, our review may not fully capture instances where thromboembolic events are significantly elevated (for example, in FRED-only cohorts), as studies solely comparing PED and FRED might inherently underrepresent these higher-risk populations. Additionally, we were unable to perform subgroup analyses based on specific device variations (PED Flex, PED Shield, and FRED X) because none of the studies reported outcomes for each device subtype separately. Considering that surface-modified FDs are considered to be safer iterations affecting the future of aneurysm treatment, comparative studies focusing on these devices are of utmost importance.

## Conclusion

The current systematic review revealed that FRED is a suitable alternative to the commonly utilized PED. Despite slight demographic differences, both devices demonstrated comparable angiographic outcomes, with similar rates of complete and adequate occlusion. In this analysis of double-arm comparative studies, complication rates and mortality were similar between the two groups except for the slightly higher hemorrhage rates observed in PED. While both PED and FRED are effective treatment options, considerations regarding adjunctive interventions and retreatment rates should be weighed when selecting the appropriate device for individual patients.

## Data Availability

No datasets were generated or analysed during the current study.

## Declarations

### Short title

PED vs. FRED for Aneurysms.
